# Evidence for a dose–response association of hyperuricemia with stroke incidence: a meta-analysis of cohort studies

**DOI:** 10.3389/fneur.2026.1900039

**Published:** 2026-08-19

**Authors:** Yuening Liu, Jie Wang, SaiWei Bai, Hong Ji

**Affiliations:** 1 School of Nursing, Shandong Second Medical University, Weifang, China; 2 School of Nursing Shandong University of Traditional Chinese Medicine, Jinan, China; 3 School of Nursing, Shandong First Medical University, Jinan, China; 4 Department of Nursing, The First Affiliated Hospital of Shandong First Medical University, Jinan, China

**Keywords:** cohort study, dose–response relationship, hyperuricemia, meta-analysis, stroke

## Abstract

**Background:**

Uric acid is considered a potential risk factor for stroke; However, whether the association between hyperuricemia and stroke outcomes follows a linear or nonlinear pattern remains unclear. Therefore, this study conducted a dose–response meta-analysis based on cohort studies to investigate the association between hyperuricemia and stroke outcomes.

**Methods:**

We systematically searched PubMed, the Cochrane Library, Embase, Web of Science, CNKI, Wanfang, VIP, and the Chinese Biomedical Literature Database from inception to April 2026. Cohort studies reporting the associations of uric acid with stroke incidence and mortality outcomes were included. Dose–response meta-analyses were performed by generalized least-squares estimation and restricted cubic splines with three knots located at the 10th, 50th, and 90th percentiles.

**Results:**

A total of 25 cohort studies involving 1,300,263 participants were included. Conventional meta-analysis showed that hyperuricemia was significantly associated with stroke incidence (RR = 1.20, 95%CI: 1.11–1.29) and mortality (RR = 1.36, 95%CI: 1.17–1.58). Subgroup analysis indicated that hyperuricemia significantly increased the risks of stroke incidence (RR = 1.22, 95%CI: 1.08–1.38) and mortality (RR = 1.42, 95%CI: 1.15–1.77) among women. Regarding stroke subtype, hyperuricemia was significantly associated with the incidence (RR = 1.21, 95%CI: 1.03–1.42) and mortality (RR = 1.23, 95%CI: 1.01–1.49) of ischemic stroke. A J shaped dose–response relationship was found between uric acid and stroke incidence (*p* = 0.0023), with risk rising across uric acid levels from104 to 764.5 μmol/L and a nadir at 225 μmol/L, whereas no significant nonlinear relationship was observed for stroke mortality.

**Conclusion:**

Hyperuricemia is associated with increased risks of stroke incidence and mortality, especially in women and ischemic stroke. A J-shaped dose–response relationship was observed for stroke incidence, but not for stroke mortality. Monitoring uric acid levels and their longitudinal changes may be valuable in stroke prevention strategies.

**Systematic Review Registration:**

https://www.crd.york.ac.uk/PROSPERO/view/CRD420261394403, identifier PROSPERO (CRD420261394403).

## Introduction

Stroke is the predominant clinical manifestation of cerebrovascular disease. According to the World Health Organization stroke fact sheet updated in December 2025 (1), stroke has become the third leading cause of death and disability worldwide, representing a major public health threat to human life, safety, and health, with a substantial disease burden. The incidence and prevalence of stroke continue to increase markedly worldwide. In 2021, approximately 93.8 million people were living with stroke globally, and approximately 11.9 million new cases occurred in the same year. Over the past two decades, the lifetime risk of stroke has increased by approximately 50%, and nearly one in four adults is estimated to experience a stroke during their lifetime. In addition, the global burden of stroke, measured by disability-adjusted life years, has shown an upward trend, increasing from 137 million DALYs in 2000 to 160 million DALYs in 2021. Stroke has therefore become a major public health issue threatening human life and health worldwide, with a heavy disease burden (2).

Although many advanced treatment approaches are currently available for stroke, its complications and sequelae have shown limited improvement. High disability and recurrence rates not only severely impair patients’ quality of life but also impose a substantial socioeconomic burden (3). Most of the stroke burden is attributable to modifiable risk factors. Therefore, strengthening prevention and improving the identification, understanding, and control of potential risk factors for stroke are of great importance for reducing disease burden and improving patient prognosis.

Uric acid is a product of purine metabolism (4) and is considered a modifiable risk factor for stroke (5, 6). Some studies have suggested that uric acid has both antioxidant and pro-oxidant properties. At physiological levels, uric acid can scavenge reactive oxygen species and peroxynitrite, thereby reducing oxidative injury to the vascular endothelium and neural tissue. However, elevated uric acid levels may increase the risk of stroke by promoting oxidative stress, inflammatory responses, endothelial dysfunction, and atherosclerosis (7). Previous studies have found that high uric acid levels may be a major risk factor for stroke.

In recent years, the association between hyperuricemia and stroke has attracted increasing attention. In addition to traditional risk factors such as hypertension, elevated fasting blood glucose, dyslipidemia, smoking, and harmful alcohol consumption, the role of metabolism-related indicators in the occurrence and progression of stroke has gradually received greater attention (8). In 2014, a review (9) on this topic concluded that hyperuricemia was associated with increased risks of stroke incidence and mortality. However, the dose–response relationship between uric acid levels and stroke outcomes has not been comprehensively evaluated. Therefore, the present review aimed to update the existing evidence by including recently published cohort studies, contribute to international academic discussion, and further clarify whether a linear or nonlinear association exists between uric acid levels and the risks of stroke incidence and mortality, thereby providing evidence to help reduce adverse stroke outcomes.

## Methods

This dose–response meta-analysis was conducted in accordance with the latest Preferred Reporting Items for Systematic Reviews and Meta-Analyses (PRISMA) guidelines (10) and was registered in the International Prospective Register of Systematic Reviews (PROSPERO registration number CRD: 420261394403) (11). The registration record described the planned review methods, including the research objective, eligibility criteria, outcomes of interest, information sources, data extraction, quality assessment, and statistical synthesis.

### Search strategy

We systematically searched PubMed, Cochrane, Embase, Web of Science, CNKI, Wanfang, VIP, and CBM from database inception to April 2026. The terms used for PubMed search are as follows: (Uric acid[MeSH Terms] OR uric acid OR urate OR hyperuricemia) AND (stroke [MeSH Terms] OR cerebrovascular accident OR Cerebrovascular Apoplexy OR Acute Stroke OR Brain Vascular Accident OR Cerebral Stroke OR Acute Cerebrovascular Accident) AND (cohort OR prospective OR follow-up OR cohort studies). In addition, after reviewing the full texts of the included studies, their reference lists were manually screened to identify additional eligible studies and minimize omissions.

### Inclusion/exclusion criteria

Studies were included if they met the following criteria: (1) The study design was a cohort study; (2) The exposure factor was uric acid; (3) The study outcomes were stroke incidence or mortality, including ischemic stroke (IS) and hemorrhagic stroke (HS); (4) The study reported clear relative risks (RR) or hazard ratios (HR) with corresponding 95% confidence intervals (CI).

The exclusion criteria were as follows: (1) Articles published as reviews, conference abstracts, letters, or case reports; (2) Studies with randomized controlled trial, cross-sectional, or case–control designs; (3) Studies in which the outcome was not stroke; (4) Studies for which the full text was unavailable or duplicate publications; and (5) Studies from which relevant effect estimates could not be extracted.

### Data extraction

Two investigators independently extracted data from each eligible article. The following information was collected from each included study: first author’s name, year of publication, country of the study population, study design, follow-up duration, stroke subtype, total number of participants, number of stroke incidence cases and death cases, uric acid levels, HR/RR, and corresponding 95% confidence intervals. Of note, all recorded deaths were attributable to stroke. The most fully adjusted estimates of HR/RR were preferentially selected for analysis. For the dose–response meta-analysis, we assigned the median or mean value of uric acid for each category to the corresponding RR for each study. If the median or mean level of each uric acid group was not available, the midpoint of the upper and lower boundaries was considered as the average level of each group. If the highest or lowest group was open-ended, half the upper bound was taken for open-ended lowest intervals and the lower bound plus half the width of the adjacent interval was applied for open-ended highest intervals.

### Quality assessment

The Newcastle–Ottawa Scale (NOS) (12) was used to assess the quality of the included cohort studies, with a maximum score of 9 points. Based on the assessment results, studies were classified as high quality (≥7 points), moderate quality (4–6 points), or low quality (≤3 points). Any disagreements were resolved through joint rereading of the original articles and discussion; when necessary, a third reviewer was consulted for assessment.

### Statistical analysis

This study used Stata 18.0 for the meta-analysis, with RR and corresponding 95%CI as the primary effect estimates. First, the units of uric acid levels reported in all studies were converted to μmol/L (1 mg/dL = 59.5 μmol/L) (13), followed by categorization. For studies reporting uric acid levels as tertiles, quartiles, or quintiles, data from the highest uric acid category or from participants with hyperuricemia, defined as uric acid ≥416 μmol/L in men and ≥357 μmol/L in women (14), were extracted for the pooled estimate of hyperuricemia. Heterogeneity among the included studies was assessed using Cochran’s *Q* test and the *I*^2^ statistic. If *p* < 0.05 and *I*^2^ > 50%, substantial heterogeneity was considered to exist among studies, and a random-effects model was used for the meta-analysis; otherwise, a fixed-effects model was applied.

Subgroup analyses were performed according to factors such as stroke subtype and sex to comprehensively explore potential sources of heterogeneity. Publication bias was assessed by visual inspection of funnel plots and Egger’s test. Sensitivity analysis was conducted using the leave-one-out method to evaluate the stability of the results.

### Dose–response meta-analysis

Stata 18.0 was used to perform the dose–response meta-analysis. We extracted the total number of participants, number of cases, RR, and corresponding 95%CI across different uric acid level categories as effect estimates. A nonlinear dose–response model was constructed to assess the association between different uric acid levels and stroke outcomes using generalized least-squares estimation and restricted cubic splines with three knots located at the 10th, 50th, and 90th percentiles (15). Nonlinearity was assessed using the Wald test. A *p* value < 0.05 was considered to indicate a nonlinear dose–response relationship between the two variables; otherwise, the relationship was considered linear. The goodness of fit of the model was also evaluated.

## Results

### Literature search

**Figure 1:**
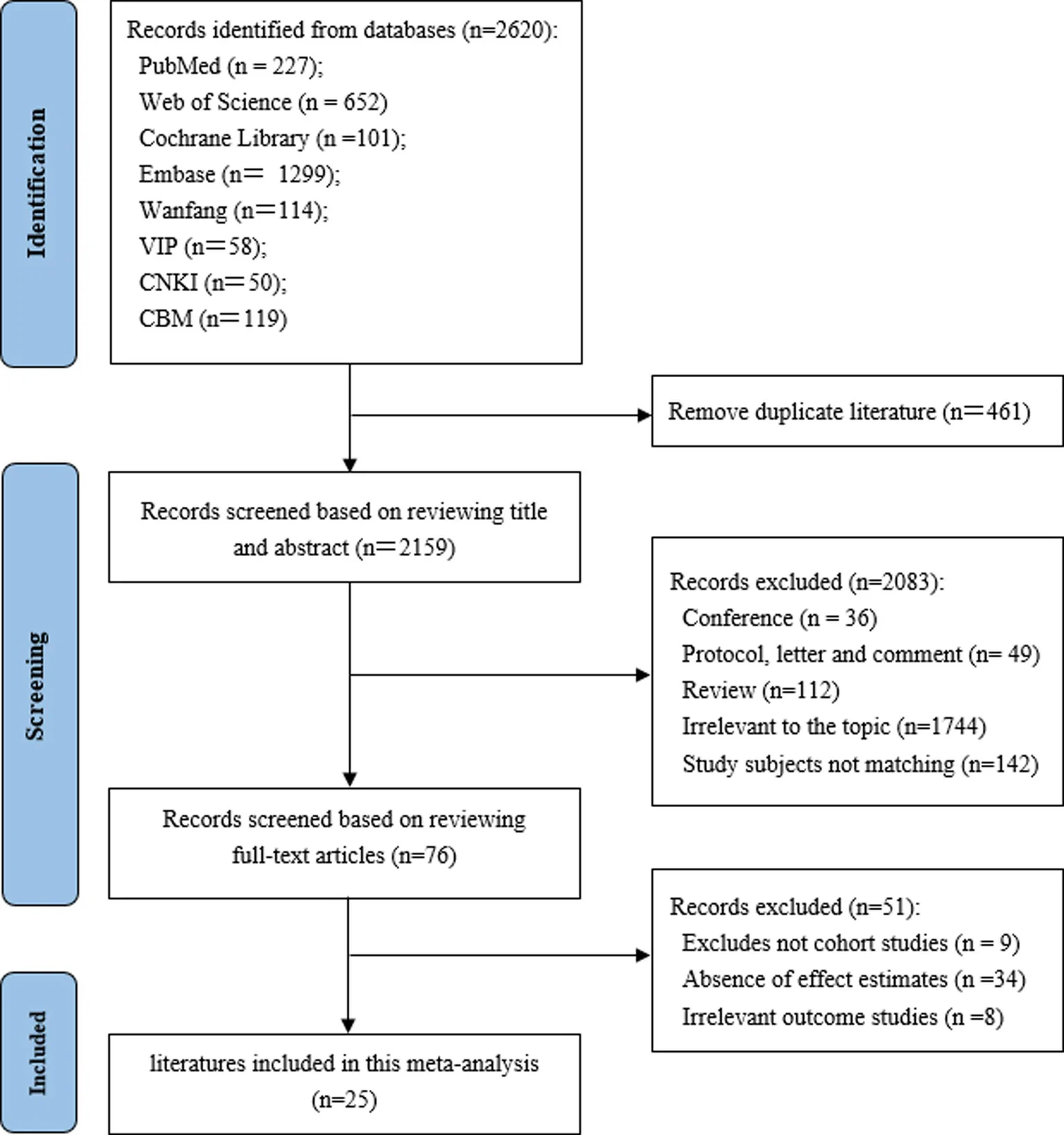
Flowchart of the selection of eligible studies.

A total of 2,620 records were identified through the literature search. After screening the abstracts and full texts, 76 articles were retained for further full-text assessment. Following further evaluation, 25 cohort studies were ultimately included. The literature screening process and results are shown in Figure 1.

### Study characteristics

The characteristics of the studies included in the meta-analysis are presented in Table 1. A total of 25 articles (16–40) were included in this study, all of which were cohort studies published between 2000 and 2025, involving 1,300,263 participants. The follow-up duration ranged from 1.68 to 23.1 years. Three studies (16, 18, 20) included only men, two studies (23, 26) included only women, and the remaining 20 studies included both men and women. Six studies (21, 25, 26, 31, 38, 40) included only ischemic stroke, whereas the remaining 19 studies included both ischemic and hemorrhagic stroke. The outcomes of interest were stroke incidence (19, 21, 22, 24, 26, 28–32, 34–38, 40) reported by 16 studies and stroke mortality (16–18, 20, 23, 25, 27, 33, 39) reported by 9 studies. One study (31) on stroke incidence and four (16, 17, 25, 39) on stroke mortality did not exclude patients with a history of stroke at baseline.

**Table 1:** Main characteristics of included studies.

First author	Country	Sex	Stroke type	Follow-up years	No. of participants	No. of cases	Outcome	Uric acid levels (μmol/L)	RR (95%CI)	NOS
Tomita M 2000 (16)	Japan	M	Stroke	5.4	49,413	127	Mortality	17.85–291.55	0.49 (0.28–0.86)	7
								297.5–380.8	1	
								386.75–499.8	1.16 (0.77–1.74)	
								505.75–773.5	2.33 (1.22–4.44)	
Mazza 2001 (17)	Italy	All	Stroke	14	3,282	170	Mortality	≤230	1.22 (1.02–1.65)	7
								240–280	1.03 (0.67–1.70)	
								290–320	1	
								330–370	1.09 (0.78–1.75)	
								≥380	1.61 (1.14–2.10)	
Jee 2004 (18)	Korea	M	Stroke	9	22,698	192	Mortality	<265	1	7
								265–306	0.97 (0.60–1.58)	
								307–354	1.03 (0.64–1.65)	
								355–414	1.35 (0.88–2.08)	
								> 414	1.10 (0.71–1.72)	
Chien 2005 (19) (Male)	China	M	Stroke	11	3,602	155	Incidence	≥458.15	1.13 (0.88–1.46)	8
Chien 2005 (19) (Female)	China	F	Stroke	11	3,602	155	Incidence	≥392.7	1.32 (1.00–1.73)	
Gerber 2006 (20)	Israel	M	Stroke	23	9,125	292	Mortality	≤232.05	1.52 (1.04–2.23)	7
								238.00–261.80	1.46 (1.00–2.12)	
								267.75–291.55	1	
								297.50–327.25	1.25 (0.85–1.84)	
								≥333.20	1.20 (0.81–1.78)	
								≤232.05	1.34 (0.87–2.05)	
								238.00–261.80	1.33 (0.89–2.01)	
								267.75–291.55	1	
								297.50–327.25	1.21 (0.81–1.82)	
								≥333.20	1.15 (0.75–1.74)	
								≤232.05	3.27 (1.14–9.33)	
								238.00–261.80	2.52 (0.87–7.29)	
								267.75–291.55	1	
								297.50–327.25	1.55 (0.49–4.89)	
								≥333.20	1.62 (0.51–5.18)	
Hozawa 2006 (21)	USA	All	IS	12.6	11,263	267	Incidence	≤285.6	1	8
								291.55–345.1	0.86 (0.60–1.23)	
								351.05–404.6	1.09 (0.79–1.49)	
								≥410.55	1.25 (0.91–1.73)	
Hozawa 2006 (21) (Male)	USA	M	IS	12.6	11,263	149	Incidence	≤285.60	1	
								291.55–345.1	1.01 (0.48–2.13)	
								351.05–404.6	1.30 (0.67–2.53)	
								≥410.55	1.63 (0.83–3.19)	
Hozawa 2006 (21) (Female)	USA	F	IS	12.6	11,263	118	Incidence	≤285.60	1	
								291.55–345.1	0.85 (0.51–1.41)	
								351.05–404.6	1.22 (0.75–1.99)	
								≥410.55	1.27 (0.70–2.30)	
Baba T 2007 (22)	Japan	All	Stroke	10	2,024	84	Incidence	≥416.5	1.07 (0.53–2.16)	7
Strasak 2008 (23)	Austria	F	Stroke	15.2	28,613	776	Mortality	≤220.15	1	9
								220.75–267.75	1.25 (0.99–1.57)	
								268.35–321.30	1.48 (1.18–1.86)	
								≥321.90	1.37 (1.09–1.74)	
			IS					≤220.15	1	
								220.75–267.75	1.17 (0.76–1.79)	
								268.35–321.30	1.19 (0.76–1.84)	
								≥321.90	1.15 (0.74–1.79)	
			HS					≤220.15	1	
								220.75–267.75	1.14 (0.65–2.01)	
								268.35–321.30	1.47 (0.83–2.52)	
								≥321.90	1.29 (0.71–2.4)	
Holme 2009 (24) (Male)	Sweden	M	Stroke	11.8	417,734	9,324	Incidence	<281	1	9
								281–319	1.03 (0.97–1.09)	
								319–362	1.09 (1.02–1.15)	
								>362	1.26 (1.19–1.34)	
			IS					<281	1	
								281–319	1.08 (1.00–1.16)	
								319–362	1.10 (1.02–1.18)	
								>362	1.30 (1.22–1.40)	
			HS					<281	1	
								281–319	0.83 (0.71–0.96)	
								319–362	0.92 (0.80–1.07)	
								>362	1.10 (0.96–1.27)	
Holme 2009 (24) (Female)	Sweden	F	Stroke	11.8	417,734	6,952	Incidence	<208	1	
								208–242	1.05 (0.97–1.15)	
								242–327	1.16 (1.07–1.26)	
								>327	1.41 (1.31–1.53)	
			IS					<208	1	
								208–242	1.12 (1.00–1.24)	
								242–327	1.27 (1.15–1.40)	
								>327	1.56 (1.42–1.72)	
			HS					<208	1	
								208–242	0.81 (0.64–1.01)	
								242–327	1.01 (0.82–1.24)	
								>327	1.13 (0.92–1.37)	
Kuo 2013 (25)	China	All	IS	4.6	354,110	2,412	Mortality	≤170	2.55 (2.09–3.11)	8
								180–290	1.33 (1.19–1.48)	
								300–410	1	
								420–530	0.96 (0.86–1.07)	
								540–650	1.04 (0.89–1.22)	
								≥660	1.22 (0.97–1.54)	
Jimenez 2016 (26)	USA	F	IS	17	32,826	460	Incidence	<232.05	1	8
								232.05–267.75	1.34 (0.91–1.97)	
								273.70–321.30	1.19 (0.80–1.75)	
								≥327.25	1.56 (1.06–2.29)	
Zhang 2016 (27) (Male)	Japan	M	Stroke	10	36,313	301	Mortality	35.70–273.70	1	9
								279.65–309.40	0.83 (0.58–1.18)	
								315.35–345.10	0.77 (0.52–1.13)	
								351.05–392.70	0.77 (0.52–1.13)	
								398.65–952.00	1.19 (0.84–1.68)	
			IS					35.70–273.70	1	
								279.65–309.40	0.87 (0.54–1.40)	
								315.35–345.10	0.75 (0.45–1.26)	
								351.05–392.70	0.91 (0.55–1.50)	
								398.65–952.00	1.19 (0.75–1.90)	
			HS					35.70–273.70	1	
								279.65–309.40	0.90 (0.46–1.77)	
								315.35–345.10	1.07 (0.54–2.14)	
								351.05–392.70	0.83 (0.41–1.68)	
								398.65–952.00	1.4 (0.75–2.65)	
Zhang 2016 (27) (Female)	Japan	F	Stroke	10	36,313	293	Mortality	23.80–196.35	1	
								202.30–226.1	1.27 (0.90–2.01)	
								232.05–255.85	0.98 (0.62–1.54)	
								261.80–297.5	1.05 (0.67–1.64)	
								303.45–636.65	1.46 (0.98–2.19)	
			IS					23.80–196.35	1	
								202.30–226.1	1.42 (0.74–2.74)	
								232.05–255.85	0.80 (0.40–1.61)	
								261.80–297.5	1.22 (0.65–2.30)	
								303.45–636.65	1.35 (0.75–2.44)	
			HS					23.80–196.35	1	
								202.30–226.1	1.41 (0.64–3.13)	
								232.05–255.85	1.33 (0.63–2.80)	
								261.80–297.5	1.09 (0.48–2.43)	
								303.45–636.65	1.54 (0.76–3.10)	
Kamei 2017 (28) (Male)	Japan	M	Stroke	2	155,322	1,089	Incidence	≤291.55	1.12 (0.92–1.37)	8
								297.5–333.2	1.07 (0.88–1.30)	
								339.15–368.9	1	
								374.85–416.5	1.00 (0.81–1.21)	
								≥422.45	1.26 (1.04–1.54)	
Kamei 2017 (28) (Female)	Japan	F	Stroke	2	155,322	992	Incidence	≤220.15	1.12 (0.90–1.38)	
								226.1–255.85	1.09 (0.89–1.33)	
								261.8–285.6	1	
								291.55–321.3	1.04 (0.85–1.29)	
								≥327.25	1.21 (1.00–1.48)	
Lazzeroni 2017 (29)	Italy	All	Stroke	2.9	1,440	32	Incidence	≥360	1.1 (0.30–2.10)	7
Shi 2017 (30)	China	All	Stroke	4.5	20,577	632	Incidence	M: <279.65 F: <226.10	1	8
								M: 279.65–327.25 F: 226.10–261.80	0.90 (0.72–1.13)	
								M: 327.25–380.80 F: 261.80–309.40	0.90 (0.71–1.13)	
								M: ≥380.80 F: ≥309.40	0.87 (0.69–1.11)	
			IS					M: <279.65 F: <226.10	1	
								M: 279.65–327.25 F: 226.10–261.80	1.01 (0.78–1.30)	
								M: 327.25–380.80 F: 261.80–309.40	0.93 (0.71–1.20)	
								M: ≥380.80 F: ≥309.40	0.95 (0.73–1.25)	
			HS					M: <279.65 F: <226.10	1	
								M: 279.65–327.25 F: 226.10–261.80	0.56 (0.32–0.97)	
								M: 327.25–380.80 F: 261.80–309.40	0.86 (0.52–1.41)	
								M: ≥380.80 F: ≥309.40	0.67 (0.38–1.16)	
Shi 2017 (30) (Male)	China	M	Stroke	4.5	20,577	300	Incidence	<279.65	1	
								279.65–327.25	0.86 (0.62–1.19)	
								327.25–380.80	0.91 (0.66–1.27)	
								≥380.80	0.80 (0.56–1.15)	
Shi 2017 (30) (Female)	China	F	Stroke	4.5	20,577	332	Incidence	<226.10	1	
								226.10–261.80	0.95 (0.69–1.31)	
								261.80–309.40	0.90 (0.65–1.24)	
								≥309.40	0.95 (0.68–1.32)	
Tscharre 2018 (31)	Austria	All	IS	5.5	1,215	39	Incidence	M: >416.50 F: >357.00	1.1 (0.49–2.06)	7
Lai 2019 (32)	China	All	Stroke	8	16,419	1,002	Incidence	M: <270 F: <214	1	8
								M: 270–316 F: 214–253	0.98 (0.81, 1.17)	
								M: 316–369 F: 253–298	0.98 (0.81, 1.18)	
								M: ≥369 F: ≥298	0.96 (0.80, 1.16)	
			IS					M: <270 F: <214	1	
								M: 270–316 F: 214–253	0.97 (0.79–1.20)	
								M: 316–369 F: 253–298	0.98 (0.80–1.21)	
								M: ≥369 F: ≥298	0.99 (0.80–1.22)	
								M: <270 F: <214	1	
								M: 270–316 F: 214–253	0.97 (0.65–1.46)	
								M: 316–369 F: 253–298	0.9 (0.64–1.45)	
								M: ≥369 F: ≥298	0.86 (0.56–1.32)	
Sakata 2020 (33)	Japan	All	Stroke	7	2,633	84	Mortality	M: 35–268 F: 95–203	3.26 (1.29–8.25)	8
								M: 269–322 F: 204–232	2.21 (0.85–5.73)	
								M: 323–357 F: 233–268	1	
								M: 358–411 F: 269–310	2.65 (1.07–6.58)	
								M: 412–690 F: 311–577	3.77 (1.54–9.24)	
Li 2020 (34) (Male)	Japan	M	Stroke	23.1	13,420	488	Incidence	41.65–273.70	1	9
								279.65–315.35	1.03 (0.78–1.36)	
								321.30–351.05	0.95 (0.71–1.27)	
								357.00–392.70	1.10 (0.82–1.48)	
								398.65–666.40	1.02 (0.74–1.35)	
			IS					41.65–273.70	1	
								279.65–315.35	1.04 (0.74–1.45)	
								321.30–351.05	0.89 (0.63–1.26)	
								357.00–392.70	1.01 (0.71–1.44)	
								398.65–666.40	1.00 (0.70–1.41)	
			HS					41.65–273.70	1	
								279.65–315.35	1.06 (0.57–1.98)	
								321.30–351.05	1.23 (0.66–2.29)	
								357.00–392.70	1.26 (0.67–2.41)	
								398.65–666.40	0.83 (0.40–1.72)	
Li 2020 (34) (Female)	Japan	F	Stroke	23.1	13,420	530	Incidence	41.65–208.25	1	
								214.20–238.00	1.02 (0.74–1.40)	
								243.95–267.75	1.20 (0.89–1.63)	
								273.70–303.45	1.15 (0.84–1.56)	
								309.40–612.85	1.45 (1.07–1.96)	
			IS					41.65–208.25	1	
								214.20–238.00	1.33 (0.88–2.02)	
								243.95–267.75	1.52 (1.02–2.26)	
								273.70–303.45	1.12 (0.73–1.72)	
								309.40–612.85	1.6 (1.07–2.41)	
			HS					41.65–208.25	1	
								214.20–238.00	0.64 (0.32–1.25)	
								243.95–267.75	0.86 (0.47–1.59)	
								273.70–303.45	1.22 (0.68–2.18)	
								309.40–612.85	1.20 (0.65–2.20)	
Chaudhary 2020 (35)	USA	All	Stroke	4	30,239	903	Incidence	<357.00	1	8
								357.00–404.60	1.3 (0.93–1.85)	
								≥404.60	1.06 (0.78–1.45)	
Chaudhary 2020 (35) (Male)	USA	M	Stroke	4	30,239	466	Incidence	<357.00	1	
								357.00–404.60	2.1 (1.29–3.45)	
								≥404.60	1.14 (0.75–1.73)	
Chaudhary 2020 (35) (Female)	USA	F	Stroke	4	30,239	437	Incidence	<357.00	1	
								357.00–404.60	0.78 (0.46–1.34)	
								≥404.60	1.04 (0.62–1.73)	
Cheng 2021 (36)	China	All	Stroke	5.78	29,974	409	Incidence	≤357	1	8
								358–374	1.07 (0.80–1.43)	
								375–395	0.85 (0.68–1.15)	
								396–427	0.78 (0.56–1.06)	
								≥428	1.27 (1.06–1.41)	
Hu 2021 (37)	China	All	Stroke	1.68	11,841	99	Incidence	38.00–325.80	1	6
								326–393.3	0.85 (0.46–1.56)	
								394–472	0.92 (0.50–1.70)	
								473–1,056	0.72 (0.36–1.44)	
			IS					38.00–325.80	1	
								326–393.3	1.01 (0.44–2.34)	
								394–472	0.73 (0.29–1.80)	
								473–1,056	0.77 (0.30–1.99)	
			HS					38.00–325.80	1	
								326–393.3	0.79 (0.14–4.49)	
								394–472	1.26 (0.25–6.38)	
								473–1,056	0.53 (0.08–3.73)	
Tan 2022 (38)	China	All	IS	4.17	5,373	240	Incidence	≤322.52	1	8
								322.53–391.77	1.79 (1.06–3.02)	
								391.78–470.01	1.84 (1.08–3.15)	
								≥470.02	1.91 (1.09–3.34)	
He 2025 (39) (Male)	USA	M	Stroke	19	32,766	813	Mortality	≥416	1.12 (0.89–1.41)	8
He 2025 (39) (Female)	USA	F	Stroke	19	32,766	813	Mortality	≥357	1.41 (1.09–1.83)	
Zhang 2025 (40)	China	All	IS	7.1	8,041	1,050	Incidence	15.47–212.42	1	7
								212.42–254.66	1.39 (1.16–1.66)	
								254.66–302.26	1.60 (1.34–1.92)	
								302.26–661.05	1.31 (1.08–1.60)	

RR, risk ratio; NOS: Newcastle–Ottawa Scale; M: male; F: female; IS: ischemic stroke; HS: hemorrhagic stroke.

The risk of bias of the included studies was assessed using the NOS. The results showed that the overall methodological quality of the included studies was high, with scores ranging from 6 to 9 points. Among them, 24 studies were rated as high quality and one study as moderate quality. The basic characteristics and risk-of-bias assessment results of the included studies are shown in Table 1.

### Meta-analysis results

#### Association between hyperuricemia and stroke incidence

**Figure 2:**
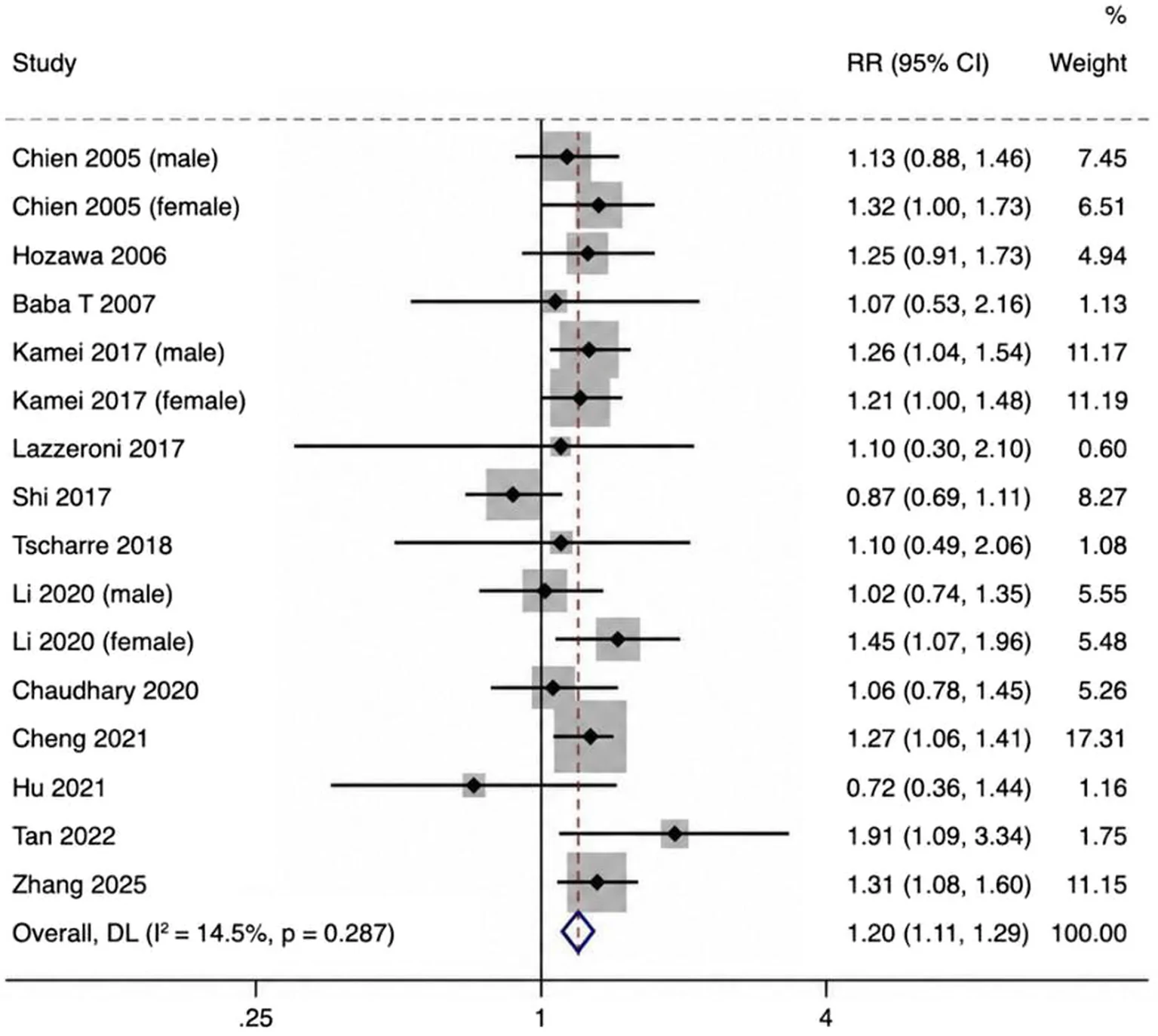
Forest plot of the association between hyperuricemia and stroke incidence.

As shown in Figure 2, 13 studies (19, 21, 22, 28–31, 34–38, 40) were included in the meta-analysis of the association between hyperuricemia and the risk of stroke incidence. No significant heterogeneity was observed among the studies (*I*^2^ = 14.5%, *p* = 0.287); therefore, a fixed-effects model was used for pooling. The pooled estimate showed a significant association between hyperuricemia and the risk of stroke incidence (RR = 1.20, 95%CI: 1.11–1.29).

#### Association between hyperuricemia and stroke mortality

**Figure 3:**
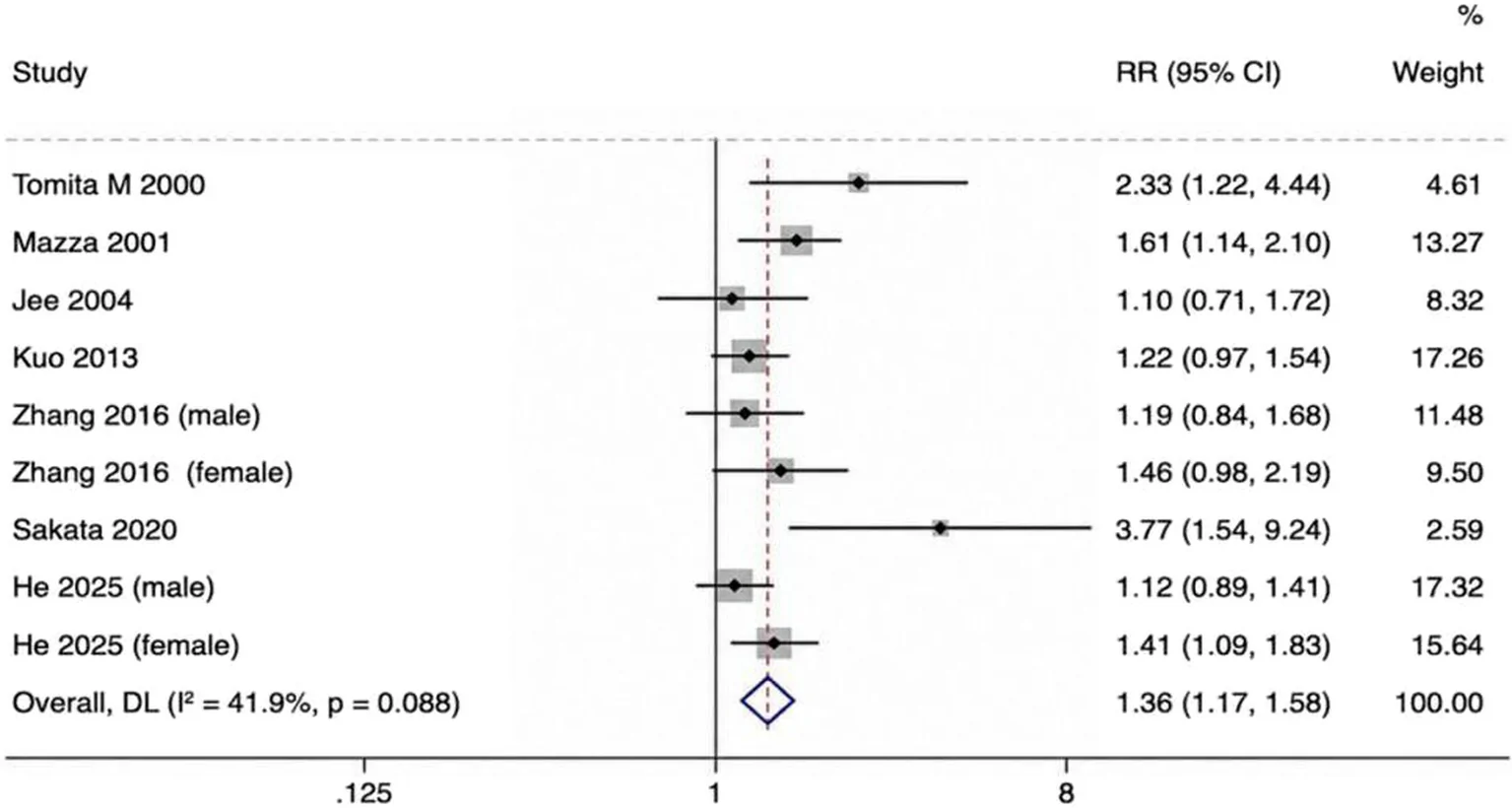
Forest plot of the association between hyperuricemia and stroke mortality.

As shown in Figure 3, seven studies (16–18, 25, 27, 33, 39) were included in the forest plot of the conventional meta-analysis examining the association between hyperuricemia and the risk of stroke mortality. No significant heterogeneity was observed among the studies (*I*^2^ = 41.9%, *p* = 0.088); therefore, a fixed-effects model was used for pooling. The pooled estimate showed a significant association between hyperuricemia and the risk of stroke mortality (RR = 1.36, 95%CI: 1.17–1.58).

#### Subgroup analyses

We performed subgroup analyses according to sex and stroke subtype, as shown in Table 2. The subgroup analysis by sex showed that hyperuricemia significantly increased the risks of stroke incidence (RR = 1.22, 95%CI: 1.08–1.38) and mortality (RR = 1.42, 95%CI: 1.15–1.77) in women. However, the associations between hyperuricemia and the risks of stroke incidence (RR = 1.12, 95%CI: 0.97–1.29) and mortality (RR = 1.23, 95%CI: 0.98–1.54) in men were not statistically significant.

**Table 2:** Subgroup analysis of the association of hyperuricemia with stroke incidence and mortality.

Groups	Incidence			Mortality		
	RR (95%CI)	*I* ^2^	*p*	RR (95%CI)	*I* ^2^	*p*
By gender						
Male	1.12 (0.97–1.29)	20.7%	0.278	1.23 (0.98–1.54)	34.0%	0.208
Female	**1.22 (1.08–1.38)**	0.0%	0.528	**1.42 (1.15–1.77)**	0.0%	0.886
By stroke type						
Ischemic stroke	**1.21 (1.03–1.42)**	32.1%	0.172	**1.23 (1.01–1.49)**	0.0%	0.942
Hemorrhagic stroke	0.85 (0.60–1.21)	0.0%	0.539	1.47 (0.92–2.35)	0.0%	0.855

Regarding stroke subtypes, the incidence of ischemic stroke was significantly associated with hyperuricemia (RR = 1.21, 95%CI: 1.03–1.42). However, no significant association was observed between hyperuricemia and the incidence of hemorrhagic stroke (RR = 0.85, 95%CI: 0.60–1.21). The association between hyperuricemia and the risk of ischemic stroke mortality was statistically significant (RR = 1.23, 95%CI: 1.01–1.49). However, hyperuricemia was not associated with the risk of hemorrhagic stroke mortality (RR = 1.47, 95%CI: 0.92–2.35).

#### Dose–response analyses

**Figure 4:**
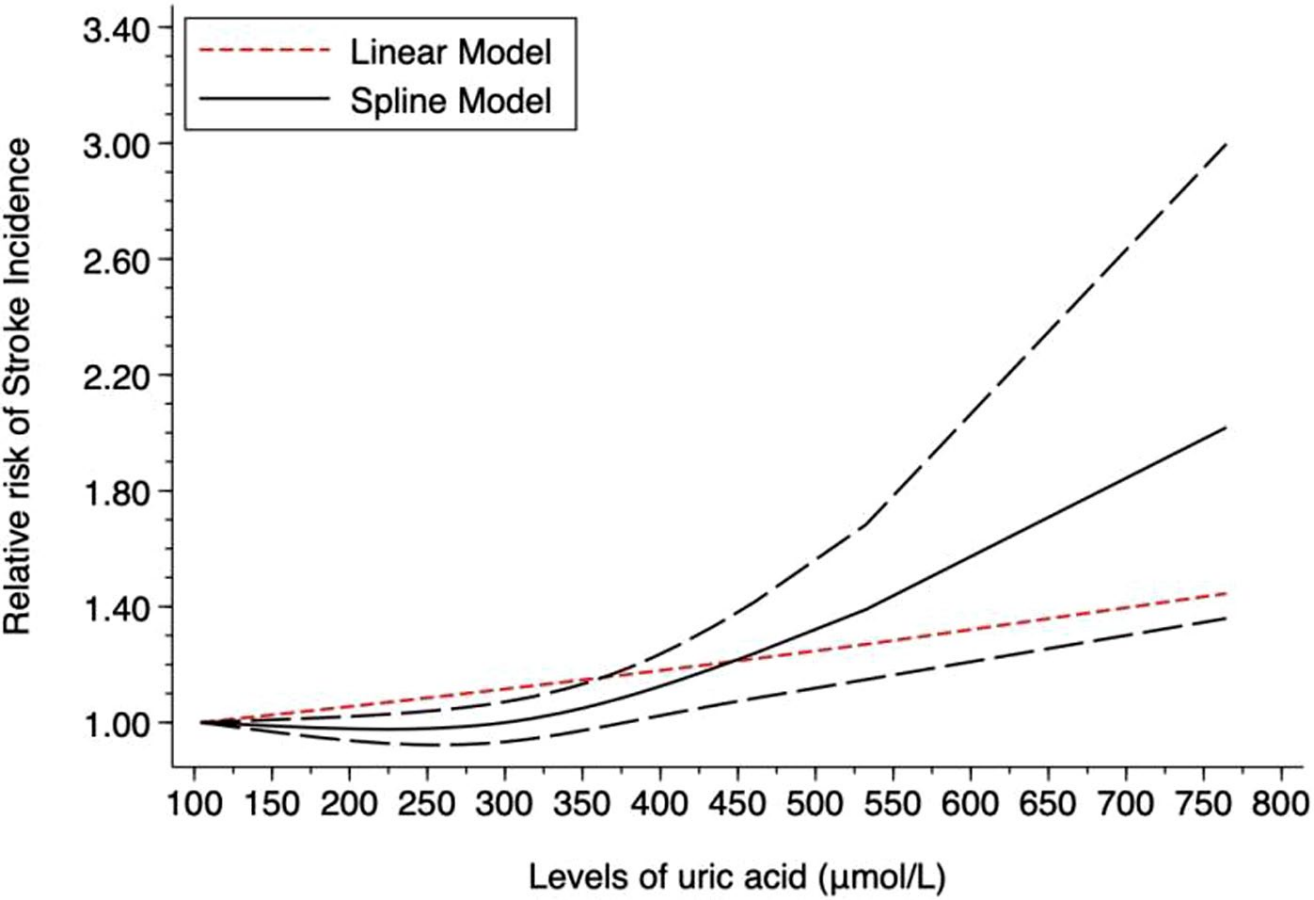
Dose–response association between serum uric acid levels and stroke incidence.

A total of 8 studies (21, 24, 26, 28, 32, 34, 36, 37) were included in the dose–response meta-analysis of the association between uric acid levels and the risk of stroke incidence. The dose–response meta-analysis showed a J-shaped relationship between elevated uric acid levels and the risk of stroke incidence (*p* = 0.0023), as shown in Figure 4. Within the serum uric acid range from 104 to 764.5 μmol/L, the risk of stroke incidence increased markedly with rising uric acid levels and the nadir of risk appeared at around 225 μmol/L.

**Figure 5:**
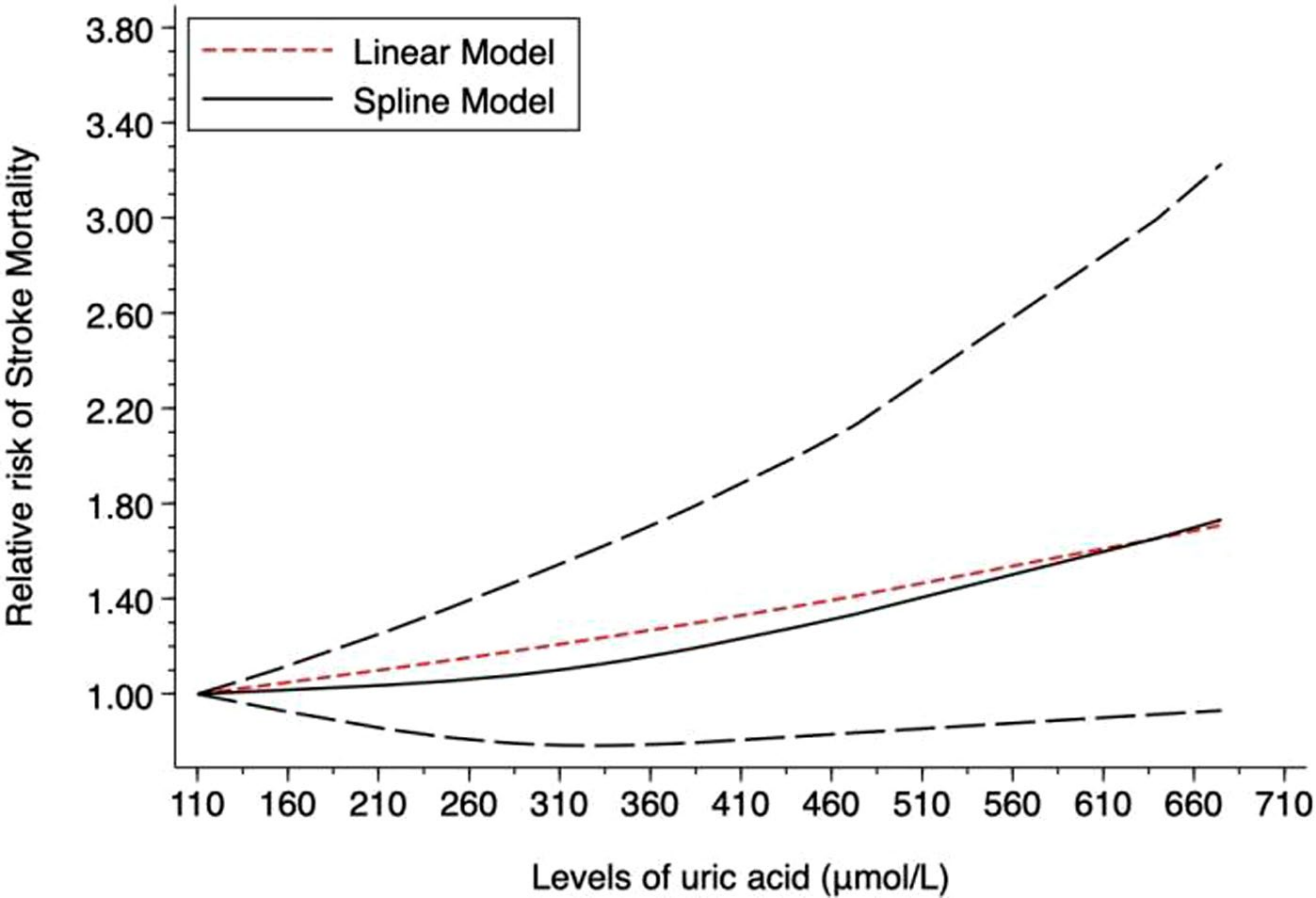
The dose–response plot on the association of serum uric acid levels with stroke mortality.

In the dose–response meta-analysis of the association between uric acid levels and the risk of stroke mortality, five cohort studies (16, 18, 20, 23, 27) provided the data required for dose–response analysis. The results showed that within the serum uric acid range from 110.1 to 675.3 μmol/L, the risk of stroke mortality increased slowly with rising uric acid levels, however the nonlinear dose–response relationship between uric acid levels and the risk of stroke mortality was not significant (*p* = 0.2384), as illustrated in Figure 5.

**Figure 6:**
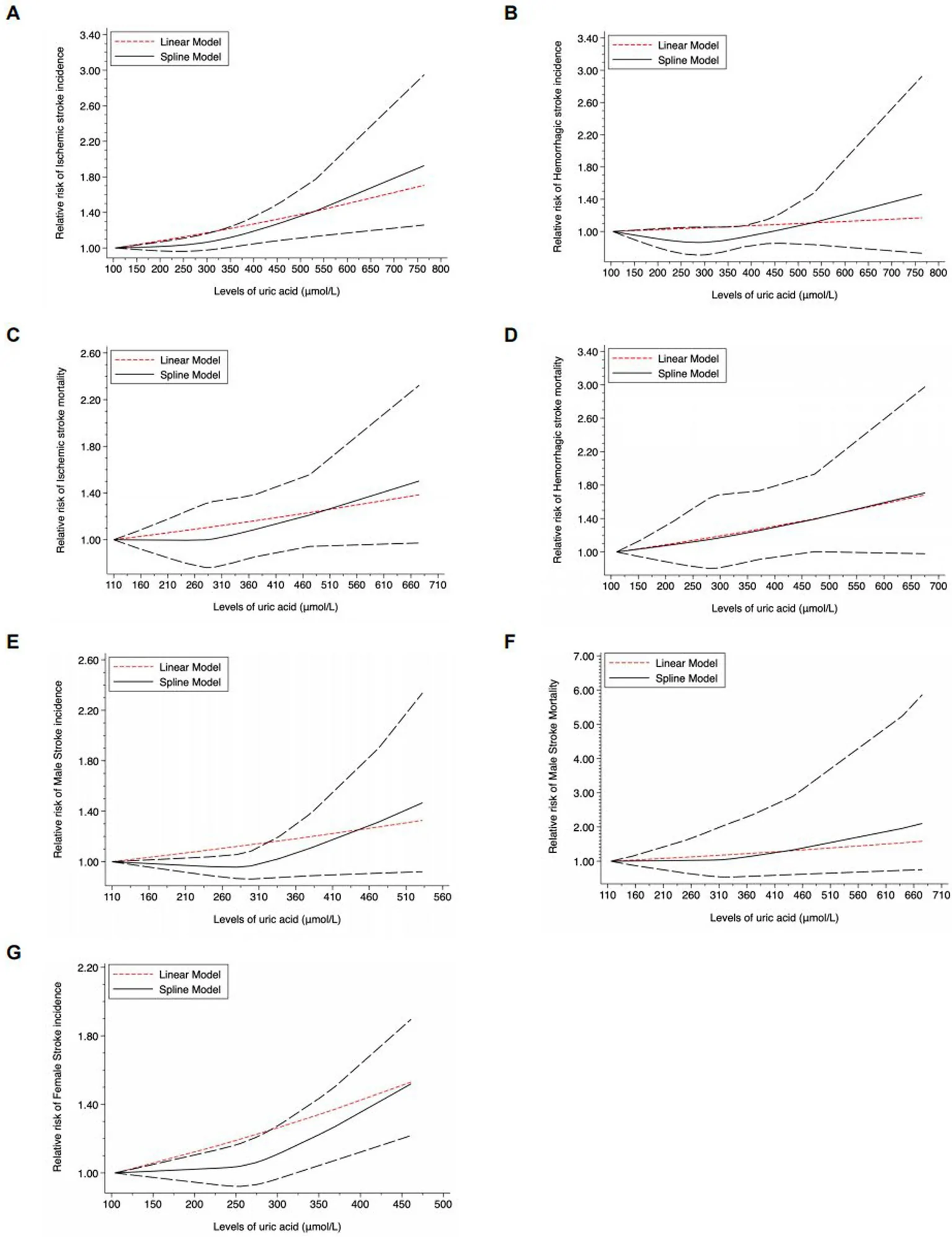
Dose–response relationship between serum uric acid levels and stroke outcomes. **(A)** Ischemic stroke incidence; **(B)** hemorrhagic stroke incidence; **(C)** ischemic stroke mortality; **(D)** hemorrhagic stroke mortality; **(E)** male stroke incidence; **(F)** male stroke mortality; **(G)** female stroke incidence.

In subgroup dose–response analyses stratified by stroke subtype, a significant dose–response relationship was observed only for uric acid level and ischemic stroke incidence (*p* = 0.0412), while the associations with ischemic stroke mortality, hemorrhagic stroke incidence, and hemorrhagic stroke mortality were not statistically significant (all *p* > 0.05), as presented in Figures 6A–D.

Subgroup dose–response analyses stratified by sex revealed an increasing trend in stroke incidence risk in both women (*p* = 0.0004) and men (*p* = 0.0034), with a mild J-shaped relationship observed in both sexes, as presented in Figures 6E,F. For stroke mortality, no significant association was found in men (*p* = 0.2409), as presented in Figure 6G; however, dose–response analysis for women was not performed due to insufficient data (only two studies).

#### Publication bias and sensitivity analysis

**Figure 7:**
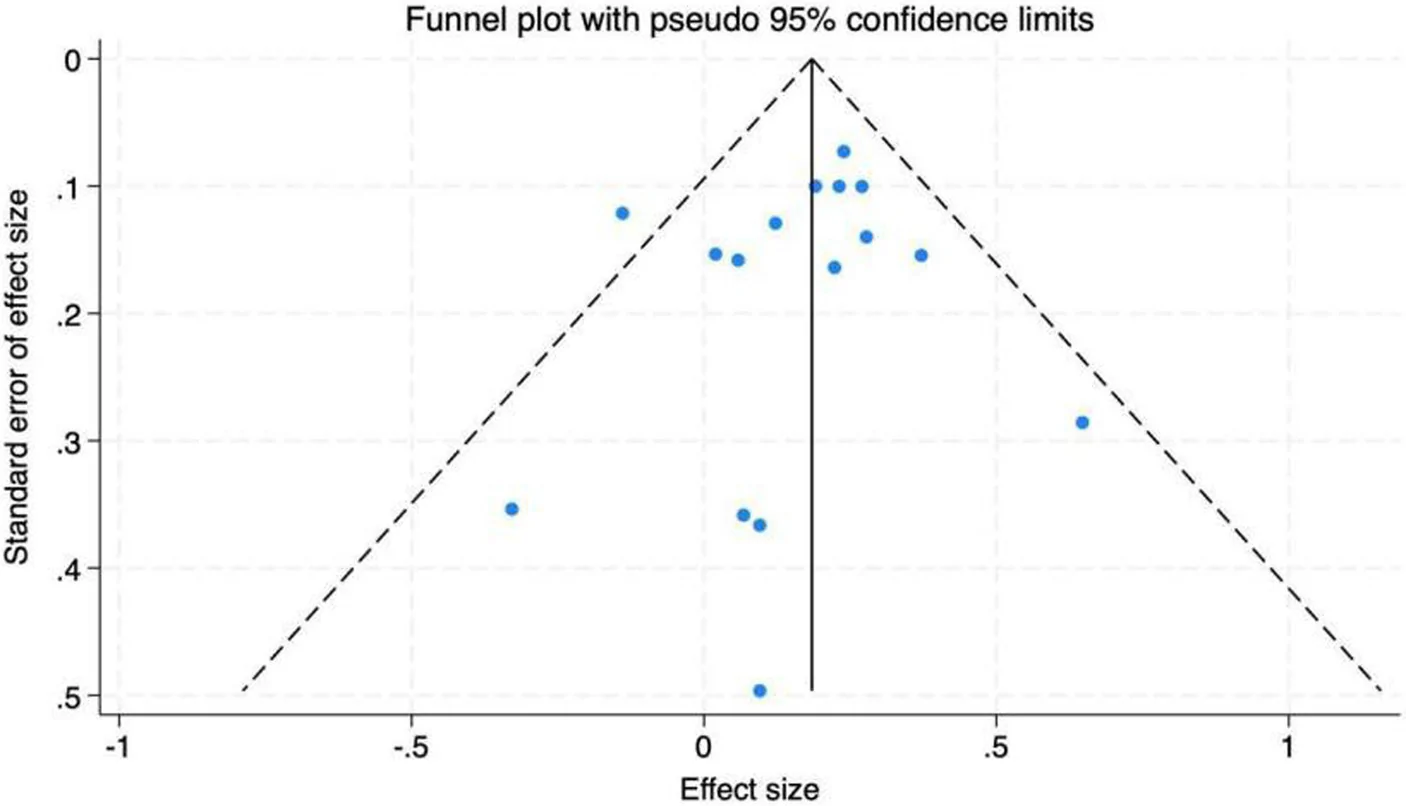
Funnel plots on the association of hyperuricemia and stroke incidence.

A funnel plot was generated to assess the association between hyperuricemia and the risk of stroke incidence. The funnel plot showed that the included studies were generally symmetrically distributed, with no obvious overall asymmetry, as shown in Figure 7.

Sensitivity analysis was performed using the leave-one-out method. Egger’s test indicated no publication bias in the association between hyperuricemia and stroke incidence (*p* = 0.491). After each individual study was sequentially excluded, the overall results showed no directional change, suggesting that the findings were stable and reliable.

## Discussion

Uric acid has dual antioxidant and pro-oxidant properties (41). At moderate levels, uric acid can act as an antioxidant by scavenging reactive oxygen species and peroxynitrite, thereby exerting certain protective effects on vascular and neural tissues. However, when uric acid levels remain persistently elevated beyond the normal range, its pro-oxidant, pro-inflammatory, and vascular-damaging effects become predominant, thereby increasing the risk of stroke. Studies have shown that high uric acid levels could cause mitochondrial calcium overload, which disrupts mitochondrial sodium-calcium exchange and subsequently leads to reactive oxygen species (ROS) production (42). A recent study (7) proposed the “uric acid oxidation–antioxidant paradox,” suggesting that uric acid may exert different effects under different physiological and pathological conditions. Experimental research (43) also indicated that uric acid can react with peroxynitrite and may have potential neuroprotective effects. Nevertheless, long-term hyperuricemia may promote oxidative stress, endothelial dysfunction, and vascular smooth muscle cell proliferation, thereby increasing the risk of stroke. In addition, a previous study (39) used NHANES data to investigate the role of inflammation in the association between hyperuricemia and stroke mortality and reported that inflammation partially mediated the relationship between hyperuricemia and mortality risk among patients with stroke. This finding provides a plausible biological explanation for the results of the present study, as persistent hyperuricemia may promote vascular injury through inflammatory pathways. However, a previous study focused on the mediating role of inflammation among patients with stroke, whereas the present meta-analysis synthesized broader cohort evidence on stroke incidence and mortality.

Previous reviews have evaluated the association between hyperuricemia and stroke, and their findings provide important context for the present study. An earlier systematic review and meta-analysis (9) published in 2014 and reported that hyperuricemia was associated with increased risks of stroke incidence and mortality. Our findings are generally consistent with those results. However, the present study extends the existing evidence by including more recently published cohort studies and updating the literature search through April 2026. In our study, 25 cohort studies were included to examine the association between hyperuricemia and stroke outcomes. A dose–response meta-analysis was further conducted to explore the potential dose–response relationship between elevated serum uric acid levels and the risks of stroke incidence and mortality. The conventional meta-analysis showed that hyperuricemia was significantly associated with stroke incidence (RR = 1.20) and stroke mortality (RR = 1.36). The dose–response meta-analysis showed a nonlinear dose–response relationship between uric acid levels and stroke incidence. A J-shaped relationship was observed between elevated uric acid levels and stroke incidence. A previous study (44) reported a nonlinear dose–response relationship between uric acid and stroke, further supporting the association between hyperuricemia and stroke outcomes. Compared with their study, the present study was not limited to the risk of stroke incidence but also included stroke mortality, which is of significance for evaluating the potential role of hyperuricemia in the disease burden of stroke.

Subgroup analyses showed that the association between hyperuricemia and stroke outcomes differed to some extent by sex and stroke subtype. The results indicated that hyperuricemia may increase the risks of stroke incidence and mortality in women. A similar finding (44) was reported that each 1 mg/dL increase in serum uric acid was associated with a more pronounced increase in stroke risk among women. The stronger association observed in women may be related to decreased estrogen levels after menopause, reduced uric acid excretion, weakened vascular protective effects, and the accumulation of risk factors such as metabolic syndrome, hypertension, and impaired renal function. When stratified by stroke subtype, the results showed that hyperuricemia was associated with the risks of ischemic stroke incidence (RR = 1.21) and mortality (RR = 1.23), whereas no significant association was observed with hemorrhagic stroke outcomes. This may be attributable to the limited number of studies and cases related to hemorrhagic stroke included in this study. These findings also suggest that hyperuricemia may contribute to the development of ischemic stroke mainly through mechanisms such as atherosclerosis, vascular endothelial injury, thrombosis, and elevated blood pressure. A recent study (24) also found that uric acid was associated with the risk of stroke and other cardiovascular outcomes. In contrast, the pathogenesis of hemorrhagic stroke is more complex and primarily involves factors such as blood pressure fluctuations, cerebral small-vessel disease, vascular wall rupture, and coagulation abnormalities (45); therefore, the role of uric acid in hemorrhagic stroke may be weaker or more strongly influenced by other factors. In addition, Şengüldür E et al. suggested that hyperuricemia may serve as a useful adjunctive parameter to support the diagnosis of suspected stroke in the emergency department, and that serum uric acid levels in at-risk patients may be valuable for patient monitoring and for guiding preventive measures (46, 47).

Although abnormal uric acid levels may serve as a risk assessment indicator, current evidence regarding the association between urate-lowering therapy and stroke outcomes remains limited. One study showed that allopurinol, a urate-lowering agent, may reduce central blood pressure and improve vascular function (48); however, these surrogate vascular indicators cannot be directly translated into reduced risks of stroke incidence or mortality. Existing clinical studies on febuxostat, another urate-lowering agent, have mainly focused on composite cardiovascular, cerebrovascular, and renal endpoints, cardiovascular safety trials, and systematic reviews (49, 50). Therefore, current evidence is insufficient to support urate-lowering therapy as an intervention for reducing the risks of stroke incidence or mortality. Further studies are needed to verify the causal relationship and potential mechanisms between urate-lowering therapy and stroke outcomes.

Our study has several strengths. First, we included 25 cohort studies involving more than 1.3 million participants. The included cohort studies had relatively long follow-up durations and large study populations, and most were of high quality, providing substantial statistical power for evaluating the association between hyperuricemia and stroke outcomes. Second, this study assessed not only the association between hyperuricemia and the risk of stroke incidence but also stroke mortality. In addition to conventional meta-analysis, a dose–response meta-analysis was performed, allowing a more in-depth examination of the nonlinear relationship between uric acid levels and stroke outcomes. Nevertheless, this meta-analysis has some limitations. First, due to the lack of necessary data in the original studies, we were unable to conduct further subgroup comparisons in the dose–response meta-analysis for stroke mortality in women. In addition, differences across studies in the timing and intervals of serum uric acid measurement may have introduced potential bias. Besides, the included populations comprised European, American, and East Asian participants, among whom baseline serum uric acid levels may differ. Finally, although fully adjusted effect estimates were preferentially extracted in this study, residual confounding could not be completely avoided, such as dietary patterns, type of hyperuricemia (primary or secondary), and difference of baseline stroke history.

## Conclusion

In summary, hyperuricemia is associated with increased risks of stroke incidence and mortality, particularly in women and for ischemic stroke. A nonlinear dose–response relationship exists between serum uric acid levels and stroke incidence and the risks of stroke incidence tended to increase with rising uric acid levels, particularly within higher uric acid ranges. Uric acid testing is simple, inexpensive, and readily available in routine health examinations and chronic disease management. In primary stroke prevention and the management of high-risk populations, attention should be paid to uric acid levels and their long-term changes. Further high-quality studies with large sample sizes are needed to determine whether urate-lowering interventions can effectively reduce the risks of stroke incidence and mortality.

## Data Availability

The original contributions presented in the study are included in the article/Supplementary material, further inquiries can be directed to the corresponding author.
