# Supplementary material for: Evidence for a dose–response association of hyperuricemia with stroke incidence: a meta-analysis of cohort studies
Source: Front Neurol. 2026 Aug 19;17:1900039. doi: 10.3389/fneur.2026.1900039 (PMC13533678; doi:10.3389/fneur.2026.1900039)
Supplement: Supplementary file 1 [file Table_1.DOCX]

**Appendix**

**Table S1. Search Strategies (Time interval: from inception to April 2026)**

| **Pubmed-227** | |
| --- | --- |
| #1 | "Uric acid"[MeSH Terms] OR "uric acid"[Title/Abstract] OR "urate"[Title/Abstract] OR "hyperuricemia"[Title/Abstract] |
| #2 | "Stroke"[MeSH Terms] OR "cerebrovascular accident"[Title/Abstract] OR "cerebrovascular apoplexy"[Title/Abstract] OR "acute stroke"[Title/Abstract] OR "brain vascular accident"[Title/Abstract] OR "cerebral stroke"[Title/Abstract] OR "acute cerebrovascular accident"[Title/Abstract] |
| #3 | "cohort studies"[MeSH Terms] OR "follow-up"[Title/Abstract] OR "prospective"[Title/Abstract] OR "cohort"[Title/Abstract] |
| #4 | #1 AND #2 AND #3 |
|  | |
| **Embase-1299** | |
| #1 | 'cerebrovascular accident'/exp |
| #2 | stroke:ab,ti OR 'cerebrovascular apoplexy':ab,ti OR 'acute stroke':ab,ti OR 'brain vascular accident':ab,ti OR 'cerebral stroke':ab,ti OR 'acute cerebrovascular accident':ab,ti |
| #3 | #1 OR #2 |
| #4 | 'uric acid'/exp |
| #5 | 'uric acid':ab,ti OR urate:ab,ti OR hyperuricemia:ab,ti |
| #6 | #4 OR #5 |
| #7 | 'cohort analysis'/exp |
| #8 | cohort:ab,ti OR prospective:ab,ti OR 'ollow up':ab,ti OR 'cohort studies':ab,ti |
| #9 | #7 OR #8 |
| #10 | #3 AND #6 AND #9 |
|  | |
| **Web of Science-652** | |
| #1 | Uric acid OR uric acid OR urate OR hyperuricemia（Topic） |
| #2 | stroke OR cerebrovascular accident OR Cerebrovascular Apoplexy OR Acute Stroke OR Brain Vascular Accident OR Cerebral Stroke OR Acute Cerebrovascular Accident（Topic） |
| #3 | cohort OR prospective OR follow-up OR cohort studies（Topic） |
| #4 | #1 AND #2 AND #3 |
|  | |
| **Cochrance library-101** | |
| #1 | MeSH descriptor: [Uric Acid] explode all trees |
| #2 | (Uric acid):ti,ab,kw OR (uric acid):ti,ab,kw OR (urate):ti,ab,kw OR (hyperuricemia):ti,ab,kw |
| #3 | #1 OR #2 |
| #4 | MeSH descriptor: [Stroke] explode all trees |
| #5 | (stroke):ti,ab,kw OR (cerebrovascular accident):ti,ab,kw OR (Cerebrovascular Apoplexy):ti,ab,kw OR (Acute Stroke):ti,ab,kw OR (Brain Vascular |
| #6 | (Cerebral Stroke):ti,ab,kw OR (Acute Cerebrovascular Accident):ti,ab,kw |
| #7 | #4 OR #5 OR #6 |
| #8 | MeSH descriptor: [Cohort Studies] explode all trees |
| #9 | (follow-up):ti,ab,kw OR (prospective):ti,ab,kw OR (cohort):ti,ab,kw |
| #10 | #8 OR #9 |
| #11 | #3 AND #7 AND #10 |
|  | |
| **China National Knowledge Infrastructure (CNKI)-50** | |
| #1 | （主题：中风 ＋ 脑中风 + 脑卒中 + 缺血性脑卒中 + 出血性脑卒 + 脑血管意外 + 脑梗死 + 脑梗塞 + 脑出血） |
| #2 | （主题：尿酸 + 高尿酸血症） |
| #3 | （主题：队列研究 + 前瞻性队列 + 回顾性队列 + 随访研究） |
| #4 | #1 AND #2 AND #3 |
|  | |
| **China BioMedical Literature Database-119** | |
| #1 | "中风"[常用字段:智能] OR "脑中风"[常用字段:智能] OR "脑卒中"[常用字段:智能] OR "缺血性脑卒中"[常用字段:智能] OR "出血性脑卒"[常用字段:智能] OR "脑血管意外"[常用字段:智能] OR "脑梗死"[常用字段:智能] OR "脑梗塞"[常用字段:智能] OR "脑出血"[常用字段:智能] |
| #2 | "尿酸"[常用字段:智能] OR "高尿酸血症"[常用字段:智能] |
| #3 | "队列研究"[常用字段:智能] OR "前瞻性队列"[常用字段:智能] OR "回顾性队列"[常用字段:智能] OR "随访研究"[常用字段:智能] |
| #4 | 1 AND #2 AND #3 |
|  | |
| **Wanfang Digital Journals-114** | |
| #1 | 主题:(中风 OR 脑中风 OR 脑卒中 OR 缺血性脑卒中 OR 出血性脑卒 OR 脑血管意外 OR 脑梗死 OR 脑梗塞 OR 脑出血) |
| #2 | 主题:(尿酸 OR 高尿酸血症) |
| #3 | 主题:(队列研究 OR 前瞻性队列 OR 回顾性队列 OR 随访研究) |
| #4 | #1 AND #2 AND #3 |
|  | |
| **VIP database for Chinese Technical Periodicals-58** | |
| #1 | 摘要=中风+脑中风+脑卒中+ 缺血性脑卒中+出血性脑卒中+脑血管意外+脑梗死+脑梗塞+脑出血 |
| #2 | 摘要=尿酸+高尿酸血症 |
| #3 | 摘要=队列研究+前瞻性队列+回顾性队列+随访研究 |
| #4 | #1 AND #2 AND #3 |
|  | |

**Table** **S2. Adjusted confounders for all studies**

| Study | Adjusted confounders |
| --- | --- |
| Tomita M 2000^[16]^ | Age, serum total cholesterol, alcohol intake, smoking, history of gout, BMI, blood glucose, systolic blood pressure, GOT, and history of cardiovascular disease. |
| Mazza 2001^[17]^ | age, historical stroke and CAD, BL IGT, arterial hypertension, systolic BP, pulse pressure, atrial fibrillation, LVHEKG, UA, status of current smoker and serum potassium and sodium. |
| Jee 2004^[18]^ | age, current smoking, diabetes, hypertension and hypercholesterolaemia. |
| Chien 2005^[19]^ | age, systolic blood pressure, body mass index, diabetes status, HDL cholesterol, LDL cholesterol, smoking and drinking history, left ventricular hypertrophy and atrial fibrillation history. |
| Gerber 2006^[20]^ | age, body mass index, systolic blood pressure, diabetes, serum cholesterol, smoking, and left ventricular hypertrophy on electrocardiogram. |
| Hozawa 2006^[21]^ | age, sex, race, education, systolic BP, diabetes mellitus, anti-hypertensive medication, cigarette smoking status, ethanol intake, serum albumin, von Willebrand factor, BMI, WHR, and low HDL cholesterol. |
| Baba T 2007^[22]^ | age, sex, smoking, and drinking, in addition to obesity and hypertension. |
| Strasak 2008^[23]^ | age, body mass index, systolic and diastolic blood pressure, total cholesterol, triglycerides, GGT, glucose, smoking status, occupational status and year of examination. |
| Holme 2009^[24]^ | age, TC, TG, hospital hypertension and diabetes status |
| Kuo 2013^[25]^ | age, sex, SUA stratum, estimated glomerular filtration rate, fasting glucose, total cholesterol and history of hypertension, diabetes mellitus, coronary heart disease, stroke, heart failure or chronic kidney disease |
| Jimenez 2016^[26]^ | age, menopausal status, postmenopausal hormone use, smoking, race/ethnicity, date of blood draw and fasting status, BMI, physical activity, alcohol intake and aspirin use， eGFR, history of diabetes and heart disease， history of hypertension， total cholesterol to HDL-C ratio and ln(hsCRP) |
| Zhang 2016^[27]^ | age, smoking status, drinking status , body mass index, triglycerides , total cholesterol, and systolic blood pressure |
| Kamei 2016^[28]^ | age, sex, obesity, hypertension, diabetes, dyslipidemia, smoking habits, alcohol consumption, eGFR, and proteinuria |
| Lazzeroni 2017^[29]^ | age, gender, arterial hypertension, diabetes, glomerular filtration rate (GFR), atrial fibrillation at discharge and medical therapy,diuretics, angiotensin-converting enzyme (ACE)-inhibitors, angiotensin receptor blockers (ARBs), aldosterone receptor antagonists (ARAs), beta-blockers and statins. |
| Shi 2017^[30]^ | sex, age, study centers, body mass index, systolic blood pressure, total cholesterol, triglycerides, glucose, eGFR, homocysteine, smoking status, and alcohol consumption at baseline and systolic blood pressure during treatment period. |
| Tscharre 2018^[31]^ | Uric acid, age, gender, body-mass index, cardiogenic shock, TIMI minor or major bleeding, traditional cardiovascular risk factors, prior MI, prior coronary revascularisation (either PCI or coronary artery bypass graft [CABG]), prior stroke or transient ischemic attack (TIA), peripheral artery disease, atrial fibrillation, heart failure, malignancy, estimated glomerular filtration rate (eGFR), peak troponin I, peak hs-CRP, serum blood glucose at admission, haematocrit at admission, vascular access site, number of affected coronary vessels, type of used stents, number of used stents, total stent length, and drug therapy (beta-blocker, ACE-I or ARB, PPI, diuretics, high-intensity statins, allopurinol and antithrombotic-therapy) |
| Lai 2019^[32]^ | age, BMI, smoking, drinking, exercise, education level, baseline hypertension, diabetes, hyperlipidemia, CVD family history, eGFR, diuretics, usual food intake frequency (poultry meat, fish and seafood, beans, vegetables and fruits) |
| Sakata 2020^[33]^ | age, sex, systolic blood pressure, antihypertensive medication, diabetes, serum total cholesterol, serum HDL cholesterol, body mass index, serum albumin, estimated glomerular filtration rate, electrocardiogram abnormalities, smoking habit, alcohol intake, and regular exercise |
| Li 2020^[34]^ | age, community, body mass index, cigarette smoking status, alcohol intake status, systolic blood pressure, atrial fibrillation, serum total cholesterol, serum triglycerides, estimated glomerular filtration rate (eGFR), diabetes mellitus, antihypertensive medication use, and in women, menopausal status |
| Chaudhary 2020^[35]^ | age, gender, race, interaction between age and race, hypertension (systolic blood pressure + diastolic blood pressure), apparent treatment-resistant hypertension, smoking, diabetes mellitus, atrial fibrillation, left ventricular hypertrophy, coronary artery disease, aspirin, lipid-lowering medications, warfarin, estimated glomerular filtration rate, and high-density lipoprotein level |
| Cheng 2021^[36]^ | age at diagnosis, gender, BMI, smoking index, lifetime total alcohol intake, diabetes status, hypertension stage, triglyceride, high-density lipoprotein, low-density lipoprotein |
| Hu 2021^[37]^ | age, sex, SBP, DBP, HR, BMI, WHR, smoking and drinking status, Hcy, TG, HDL-C, LDL-C, eGFR, total bilirubin, ALT, DM, and antiplatelet agents. |
| Tan 2022^[38]^ | age, sex, BMI, diabetes history, SBP, DBP, smoking, drinking, waist circumference, fasting blood glucose, TG, LDL-C, HDL-C, TP, diuretic, antihypertensive drug, hypoglycemic drug, lipid-lowering drug, uric acid-lowering drug, eGFR |
| He 2025^[39]^ | sex, age, race, poverty level, marital status, education level, smoking, drinking, hypertension, coronary heart disease, diabetes, cancer, body mass index, total cholesterol, aspartate aminotransferase, alanine aminotransferase, albumin, monocytes, neutrophils, lymphocytes, and platelets |
| Zhang 2025^[40]^ | age, sex, marital status, residence, education level, smoking, drinking, hypertension, dyslipidemia, diabetes, and coronary heart disease |

**Table S3. Example of dose assignment for uric acid categories in the Hozawa 2006 study**

| **Uric acid levels (µmol/L)** | **Dose assignment method** | **Representative dose (µmol/L)** |
| --- | --- | --- |
| ≤285.6 | 285.6 × 0.5 | 142.8 |
| 291.55–345.1 | (291.55 + 345.1) / 2 | 318.3 |
| 351.05–404.6 | (351.05 + 404.6) / 2 | 377.8 |
| ≥410.55 | 410.55 + 0.5 × (404.6 − 351.05) | 437.3 |

**Table S4. The association between uric acid levels and stroke incidence risk in dose-response meta-analysis**

| dose | RR | lbs | ubs |
| --- | --- | --- | --- |
| 104 | 1 | 1 | 1 |
| 110.1 | 0.99843 | 0.995657 | 1.001212 |
| 116 | 0.996914 | 0.991474 | 1.002385 |
| 125 | 0.994606 | 0.985127 | 1.004177 |
| 140.5 | 0.990644 | 0.974291 | 1.007271 |
| 142.8 | 0.990057 | 0.972693 | 1.007731 |
| 145.8 | 0.989294 | 0.970616 | 1.008333 |
| 157.7 | 0.986327 | 0.962495 | 1.01075 |
| 178.5 | 0.981663 | 0.949176 | 1.01526 |
| 181.9 | 0.980999 | 0.947154 | 1.016053 |
| 204.2 | 0.977647 | 0.935381 | 1.021823 |
| **225** | **0.976583** | **0.927349** | **1.028431** |
| 226.1 | 0.976595 | 0.927019 | 1.028822 |
| 241 | 0.977535 | 0.923593 | 1.034628 |
| 249.9 | 0.978856 | 0.922548 | 1.038601 |
| 255.9 | 0.980099 | 0.922297 | 1.041524 |
| 260.1 | 0.98115 | 0.922349 | 1.043699 |
| 273.7 | 0.985647 | 0.923866 | 1.051559 |
| 284.5 | 0.990513 | 0.92661 | 1.058823 |
| 288.6 | 0.992686 | 0.928024 | 1.061853 |
| 297.5 | 0.998061 | 0.931817 | 1.069014 |
| 297.5 | 0.998061 | 0.931817 | 1.069014 |
| 300 | 0.999739 | 0.933063 | 1.071181 |
| 303.5 | 1.002219 | 0.934941 | 1.074339 |
| 306.4 | 1.004391 | 0.936617 | 1.07707 |
| 315.4 | 1.011818 | 0.942483 | 1.086253 |
| 318.3 | 1.014427 | 0.944572 | 1.089448 |
| 336.2 | 1.032756 | 0.959245 | 1.111901 |
| 340.5 | 1.037706 | 0.963148 | 1.118035 |
| 351.1 | 1.05076 | 0.973231 | 1.134466 |
| 351.1 | 1.05076 | 0.973231 | 1.134466 |
| 354 | 1.054536 | 0.976083 | 1.139295 |
| 359.7 | 1.062205 | 0.98178 | 1.149218 |
| 366 | 1.071049 | 0.988187 | 1.160859 |
| 369.5 | 1.076124 | 0.991785 | 1.167636 |
| 374.9 | 1.084173 | 0.997372 | 1.178529 |
| 377.8 | 1.088602 | 1.000386 | 1.184598 |
| 383.5 | 1.097517 | 1.006325 | 1.196973 |
| 385 | 1.099908 | 1.007889 | 1.200328 |
| 386.2 | 1.101833 | 1.00914 | 1.203041 |
| 395.7 | 1.117476 | 1.019035 | 1.225427 |
| 411.5 | 1.144922 | 1.035329 | 1.266115 |
| 433 | 1.184632 | 1.056919 | 1.327777 |
| 437.3 | 1.192833 | 1.061135 | 1.340877 |
| 443.3 | 1.20439 | 1.066954 | 1.35953 |
| 443.5 | 1.204778 | 1.067147 | 1.360159 |
| 461.1 | 1.239351 | 1.0838 | 1.417226 |
| 532.5 | 1.390085 | 1.148031 | 1.683175 |
| 764.5 | 2.018413 | 1.359307 | 2.99711 |

**Table S5. The association between uric acid levels and stroke mortality risk in dose-response meta-analysis**

| dose | ors | lbs | ubs |
| --- | --- | --- | --- |
| 110.1 | 1 | 1 | 1 |
| 116 | 1.001975 | 0.990767 | 1.01331 |
| 132.5 | 1.007519 | 0.965396 | 1.05148 |
| 154.7 | 1.015026 | 0.932282 | 1.105114 |
| 214.2 | 1.037145 | 0.852495 | 1.261791 |
| 244 | 1.05168 | 0.821388 | 1.346539 |
| 244.3 | 1.051845 | 0.821114 | 1.347411 |
| 249.9 | 1.055009 | 0.816169 | 1.363742 |
| 279.7 | 1.07477 | 0.795285 | 1.452473 |
| 285.5 | 1.079275 | 0.792369 | 1.470065 |
| 294.5 | 1.086754 | 0.788645 | 1.497548 |
| 294.8 | 1.087014 | 0.788538 | 1.498468 |
| 312.4 | 1.103481 | 0.784138 | 1.552877 |
| 330.2 | 1.122447 | 0.783047 | 1.608954 |
| 330.5 | 1.122785 | 0.783054 | 1.609909 |
| 339.2 | 1.132865 | 0.783607 | 1.637789 |
| 348.1 | 1.143685 | 0.784809 | 1.666667 |
| 371.9 | 1.174965 | 0.790717 | 1.745937 |
| 384.5 | 1.192784 | 0.79514 | 1.789286 |
| 443.3 | 1.28478 | 0.822226 | 2.007552 |
| 443.5 | 1.285112 | 0.822325 | 2.008346 |
| 473 | 1.334928 | 0.836764 | 2.129673 |
| 639.6 | 1.654767 | 0.914136 | 2.995456 |
| 675.3 | 1.732707 | 0.930168 | 3.227667 |
